# Enhanced prediction of ventilator-associated pneumonia in patients with traumatic brain injury using advanced machine learning techniques

**DOI:** 10.1038/s41598-025-95779-0

**Published:** 2025-04-02

**Authors:** Negin Ashrafi, Armin Abdollahi, Kamiar Alaei, Maryam Pishgar

**Affiliations:** 1 Department of Industrial and Systems Engineering, University of Southern California, Los Angeles, CA USA; 2 Department of Electrical and Computer Engineering, University of Southern California, Los Angeles, CA USA; 3 Department of Health Science, California State University, Long Beach (CSULB), Long Beach, CA USA

**Keywords:** Data mining, Machine learning, Statistical methods, Computer science, Diseases

## Abstract

Ventilator-associated pneumonia significantly increases morbidity, mortality, and healthcare costs among patients with traumatic brain injury. Accurately predicting risk can facilitate earlier interventions and improve patient outcomes. This study leveraged the MIMIC III database, identifying traumatic brain injury cases through standardized clinical criteria. A rigorous data preprocessing workflow included missing value imputation, correlation checks, and expert-driven feature selection, reducing an initial set of features to a subset of critical predictors encompassing demographics, comorbidities, laboratory values, and clinical interventions. To address class imbalance, the Synthetic Minority Oversampling Technique (SMOTE) was applied within a five-fold cross-validation framework, ensuring a balanced training set while maintaining an unbiased validation process. Six machine learning models, including Support Vector Machine, Logistic Regression, Random Forest, XGBoost, Artificial Neural Network, and AdaBoost, were trained using extensive hyperparameter tuning. Comprehensive evaluations were conducted based on multiple metrics, including Area Under the Curve (AUC), accuracy, F1 score, sensitivity, specificity, Positive Predictive Value, and Negative Predictive Value. XGBoost emerged as the top performing algorithm, achieving an AUC of 0.94 and an accuracy of 0.875 on the test set, marking substantial improvements over previously reported best results. An ablation study validated the necessity of each retained feature, indicating that any feature removal led to a decline in model performance. Furthermore, SHAP analysis underscored ICU length of stay, hospital length of stay, serum potassium, and blood urea nitrogen as key contributors to ventilator associated pneumonia risk. Overall, the results demonstrate that advanced ensemble learning, meticulous feature selection, and effective class imbalance handling can significantly enhance early detection in traumatic brain injury cases. These findings have meaningful clinical implications, offering a framework for more timely interventions, optimized resource allocation, and improved patient care in critical settings.

## Introduction

Traumatic brain injury (TBI) affects approximately 250 per 100,000 individuals globally, contributing to 30-50% of trauma-related mortalities, with adolescents, young adults, and older adults being the most affected groups^1,2^. TBI occurs from forceful impacts to the head or body, resulting in varying degrees of cognitive and physical impairment^3^. Complicating the management of TBI is the frequent occurrence of ventilator-associated pneumonia (VAP), a lung infection that develops in patients requiring mechanical ventilation. VAP not only exacerbates the morbidity and mortality associated with TBI but also prolongs hospital stays and increases healthcare costs^4^. Therefore, early identification and prediction of VAP in TBI patients are crucial for improving patient outcomes and reducing the burden on healthcare systems.

Several studies have investigated the prediction and management of VAP in TBI patients, leveraging diverse methodologies and datasets to enhance our understanding of this complex clinical scenario. Wang et al. utilized machine learning techniques to predict VAP in patients with TBI, leveraging data from the Medical Information Mart for Intensive Care III (MIMIC-III) database. Their study explored various models, including XGBoost, SVM, Logistic Regression, and Random forest, emphasizing the importance of identifying pertinent features for accurate prediction^5^. Robba et al., on the other hand, conducted a large-scale prospective observational study to analyze the incidence, risk factors, and outcomes of VAP in TBI patients. Their findings underscored the multifactorial nature of VAP development, highlighting age and smoking as potential risk factors influencing patient susceptibility^6^.

Luo et al. investigated the impact of VAP on the prognosis of ICU patients within 90 and 180 days, identifying critical factors such as diabetes, length of ICU stay, and COPD that influence patient outcomes. Their study also proposed methodologies for enhancing diagnostic approaches in identifying VAP patients^7^. Recent studies have shown significant improvements in the power of predicting mortality in mechanically ventilated ICU patients using advanced techniques and enhanced feature selection^8^. Additionally, Texture-based Gabor features have proven effective in identifying COVID-19 stages from CT images^9^, and similar methods have been used to classify lung diseases via Littlewood Paley empirical wavelet transforms^10^. Such imaging-driven approaches highlight the versatility of ML in healthcare, complementing EHR-focused models by revealing additional clinical insights from radiological data.

Collectively, these studies underscore the importance of predictive modeling, risk factor analysis, and feature selection in improving the identification and management of VAP in TBI patients. Machine learning algorithms offer promising avenues for predicting VAP development while understanding risk factors and prognosis aids in personalized patient care. Further research integrating diverse methodologies and datasets is warranted to enhance the accuracy and generalizability of predictive models in this critical domain.

Despite these advancements, the interplay between TBI severity and the emergence of VAP remains multifaceted. Prior research indicates that factors such as the timing of mechanical ventilation initiation, neurosurgical interventions, and the patient’s immune response all contribute to infection susceptibility^11,12^. Moreover, prolonged ICU stays-often inherent to TBI management-further compound the risk for VAP through increased exposure to invasive procedures and multidrug-resistant pathogens^13^. Understanding these clinical complexities is imperative for developing more nuanced predictive models and targeted therapeutic strategies.

Additionally, recent work has emphasized the potential of sophisticated machine learning algorithms for capturing complex, non-linear relationships in clinical data^14,15^. Notably, advanced ensemble techniques, deep neural architectures, and interpretable AI methods (e.g., SHAP analysis) have emerged as powerful tools for prognostication and risk stratification in critical care^16,17^. However, challenges remain in optimizing feature selection, handling class imbalance, and ensuring clinical interpretability, particularly within heterogeneous datasets like MIMIC-III. By refining these approaches and integrating expert domain knowledge, researchers aim to build more accurate, explainable models that not only flag at-risk patients but also provide actionable insights for early intervention and improved patient outcomes^18,19^. The main contributions of this study are as follows:

- We introduce an advanced feature selection approach that reduces the original 52 candidate features to the 15 most critical ones using CatBoost importance scores, supported by clinical expert validation.
- Our methodology effectively handles class imbalance by integrating SMOTE within a rigorous 5-fold cross-validation framework, which enhances the robustness of our predictive model.
- A comprehensive model evaluation-including detailed analysis of the confusion matrix, ROC curves, and a systematic ablation study-demonstrates significant performance improvements, with an AUC of 0.94 and an accuracy of 0.875, outperforming previous studies.
- We employ SHAP analysis to elucidate the contribution of key features (e.g., ICU stay length, hospital stay length, serum potassium, and blood urea nitrogen), thereby improving the interpretability of our model and supporting its clinical applicability.

## Methodologies

### Data source and study design

In this study, we employed the MIMIC-III database, a publicly available clinical dataset that includes detailed medical information from over 40,000 patients^20^. These patients were treated at two prominent hospitals in the United States between 2001 and 2012. The dataset encompasses over 60,000 hospital admissions and more than 20,000 intensive care unit (ICU) stays, amounting to more than 40 million individual clinical record entries. These entries cover a wide range of data points, including physiological signals, medication administrations, laboratory test results, and clinical notes. The extensive scope and detailed nature of MIMIC-III make it an invaluable resource for conducting rigorous biomedical research, enabling us to thoroughly analyze and validate our methodologies within a clinical setting.

### Patient selection for TBI-VAP analysis

**Fig. 1:**
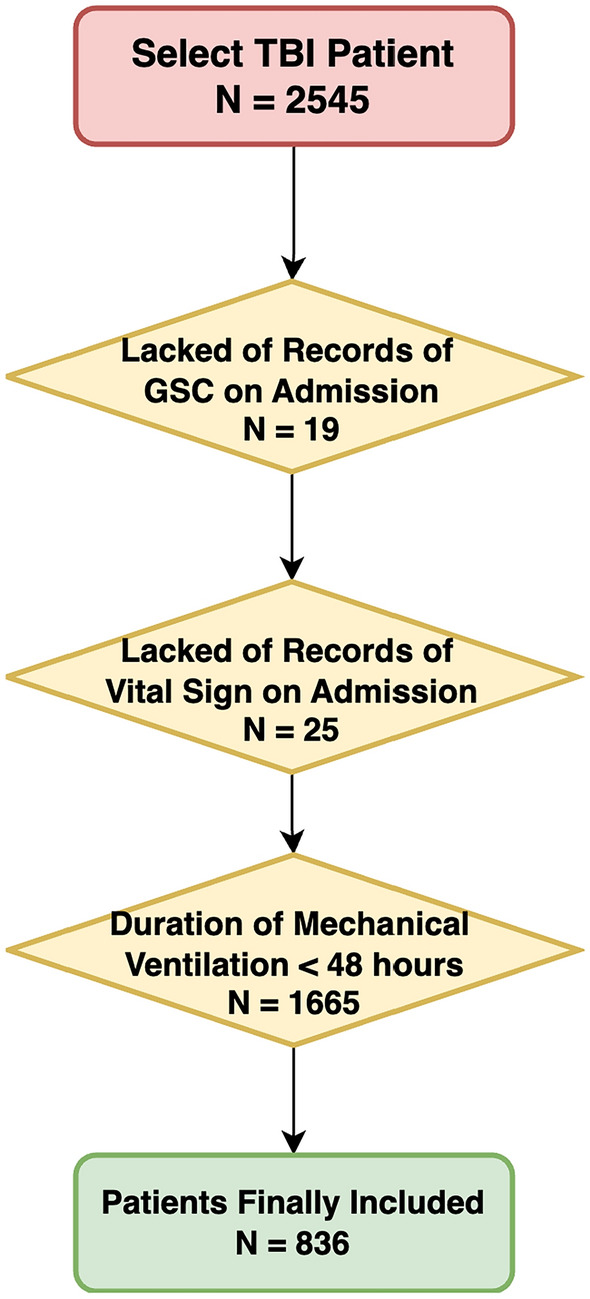
Flow diagram of the patient selection process.

To identify TBI patients for our study, specific International Classification of Diseases and Ninth Revision (ICD-9) codes were utilized. Patients were initially selected based on the following codes: 80,000-80,199; 80,300-80,499; 8500-85419, which identified an initial cohort of 2545 patients^21^. Exclusions were applied for patients lacking Glasgow Coma Scale (GCS) records at admission (19 patients) or those without recorded vital signs at admission (25 patients). Further exclusions were made for patients who underwent mechanical ventilation for less than 48 hours, impacting 1665 patients. After all exclusions, the final cohort consisted of 836 patients. Within this group, 328 were identified as positive for VAP, while 508 were negative. This methodology ensures a focused examination of the impact of TBI on the risk of VAP, guided by our predefined clinical criteria. Although these criteria narrowed our sample from 2545 to 836 patients, they ensured that each case had complete, high-quality data on TBI severity, vital signs, and ventilation duration-factors crucial for robust modeling of VAP risk. In particular, the 48-hour threshold was chosen to capture a clinically significant window for VAP onset, thus enhancing the dataset’s relevance and reliability for our predictive analysis. Figure 1 shows the patient extraction process

### Statistical analysis

The differences between train and test cohorts were assessed using two-sided t-tests. This test choice is appropriate under the assumption that the data approximately follow a normal distribution, though it is robust to mild deviations from this assumption. For binary or categorical variables like Tracheostomy and Neurosurgery, a Chi-Square test was employed to determine if the distribution of categories differs significantly between the cohorts^22^. This test is chosen assuming that the observed frequencies are large enough to meet the test’s conditions.

### Feature engineering and selection

In our study, the target variable, VAP, is identified through a structured diagnostic approach that encompasses three key criteria: radiologic, systemic, and pulmonary signs. (1) For radiologic confirmation, a patient must show at least one of the following: new or progressive and persistent infiltrate, consolidation, or cavitation on lung imaging, indicative of the physical manifestations of pneumonia. (2) Systemically, the criteria require either a fever exceeding 38 °C or an abnormal white blood cell count, with thresholds set below 4,000/mL or above 12,000/mL, signaling an immune response to infection. (3) On the pulmonary front, a diagnosis is supported by the presence of at least two symptoms: purulent sputum, deteriorating gas exchange, and worsening of respiratory symptoms such as cough, dyspnea, tachypnea, or new breath sounds^23^. This multidimensional diagnostic framework ensures a thorough and precise identification of VAP in critically ill patients, capturing the complex clinical profile necessary for accurate diagnosis and subsequent treatment planning. This is summarized in 1.

**Algorithm 1:**
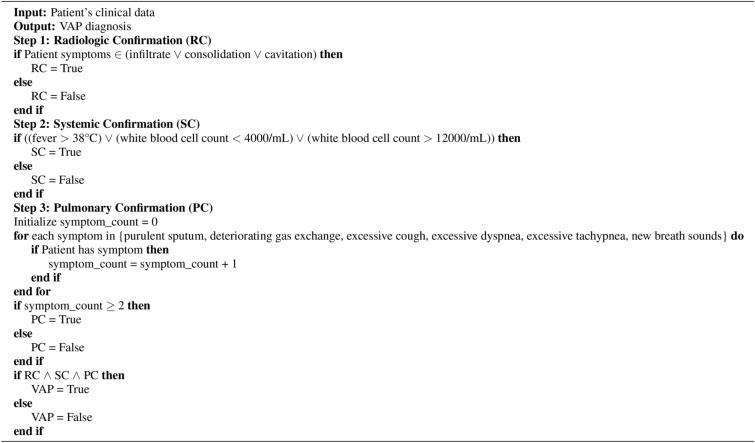
Diagnostic Algorithm for Identifying VAP

37 candidate features were meticulously chosen to capture a broad spectrum of clinical information crucial for assessing the patients’ health outcomes. These features are categorized into demographic details, disease-related attributes, vital signs at admission, intracranial injury classifications, laboratory tests, medical interventions, and other relevant clinical data. The demographic features include age, gender, and ethnicity, which are foundational for adjusting clinical analyses. Disease-related features such as smoking history, diabetes, hypertension, and other significant conditions are included due to their potential impact on patient prognosis. Vital signs such as heart rate and blood pressure, recorded at admission, provide immediate clinical context. Intracranial injury features, including conditions like subarachnoid hemorrhage and subdural hematoma, are particularly critical for patients with traumatic brain injuries. Laboratory tests, including measurements of platelet count, hemoglobin levels, and blood urea nitrogen, among others, offer detailed biochemical insights that are indispensable for monitoring patient health and response to treatment. Medical interventions like tracheostomy and neurosurgery, recorded to capture the intensity and nature of the medical care provided, also contribute to understanding patient outcomes. The selection process was meticulously guided by consultations with a clinical expert and a comprehensive review of the existing literature. This rigorous approach ensured that each feature included in the study was highly relevant to the clinical questions posed, as a result significantly enhancing the robustness and practical applicability of our predictive models. This thoughtful compilation of features enables a holistic view of patient health, supporting the identification of patterns and predictors of recovery in a clinically diverse population.

**Fig. 2:**
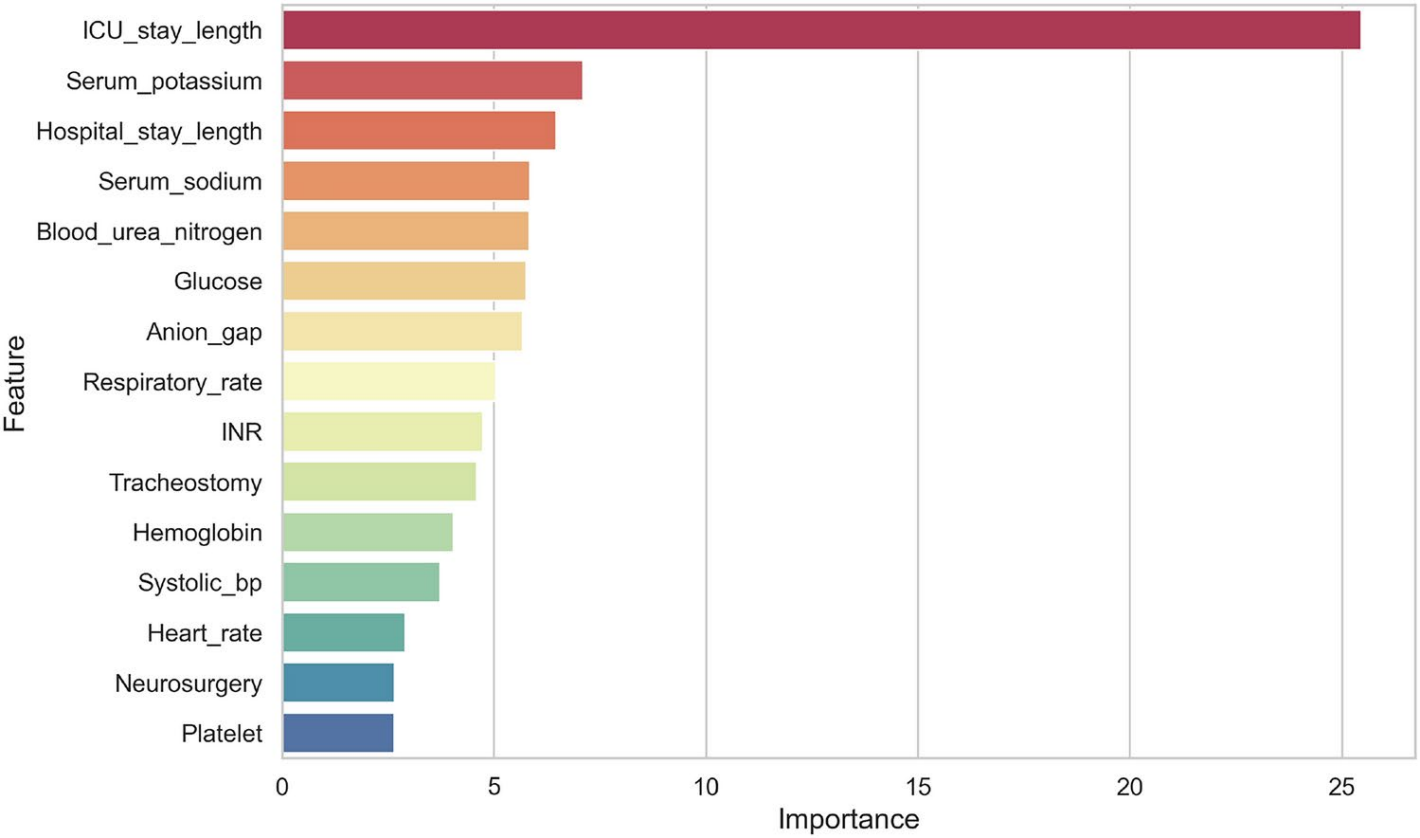
Top 15 features based on CatBoost feature importance scores, highlighting the most impactful ones.

To refine the feature set, we first applied a correlation matrix to identify and remove highly correlated features, thereby reducing redundancy within our dataset. This process led to the exclusion of features such as PEG (percutaneous endoscopic gastrostomy), serum chloride, and red blood cell count, which were found to provide overlapping information. Subsequently, we employed the CatBoost algorithm to determine the relative importance of the remaining features, guiding us to focus on those most influential for our predictive model. The top 15 features, as identified by CatBoost, include critical indicators such as ICU stay length, serum potassium, and hospital stay length, among others, as shown in the accompanying feature importance chart, which is shown in Fig. 2. These features were selected for their strong predictive power and clinical relevance, based on expert opinion, ensuring that our model is both accurate and interpretable in assessing patient outcomes.

### Data preprocessing

In the preprocessing stage of our data analysis, we implemented systematic approaches to manage missing values, encode categorical data, and scale features appropriately. For numerical data, missing values were imputed with the median of each respective feature, given its robustness to outliers, thereby maintaining the integrity of the data distribution. For categorical data, missing entries were filled using the mode, ensuring the most frequent category was used for a consistent and representative fill. Further enhancing our model’s ability to process categorical variables, one-hot encoding was applied. We opted for a simple median (or mode) imputation strategy to manage missing values, primarily due to relatively small fraction of missing data in our dataset. For larger or more complex datasets with substantial missingness, multiple imputation would indeed be preferable.

In terms of feature scaling, different strategies were employed based on the specific requirements of the algorithms used in subsequent analyses. For models sensitive to the scale of input features, a min-max scaler was utilized to normalize the data within a range of 0 to 1^24^. This scaling preserves the relationships among the original data points. For other algorithms, we applied a standard scaler, which standardized features by removing the mean and scaling to unit variance. This approach is particularly beneficial for models that assume data is normally distributed, such as many linear models, and helps in reducing the influence of outliers.

**Fig. 3:**
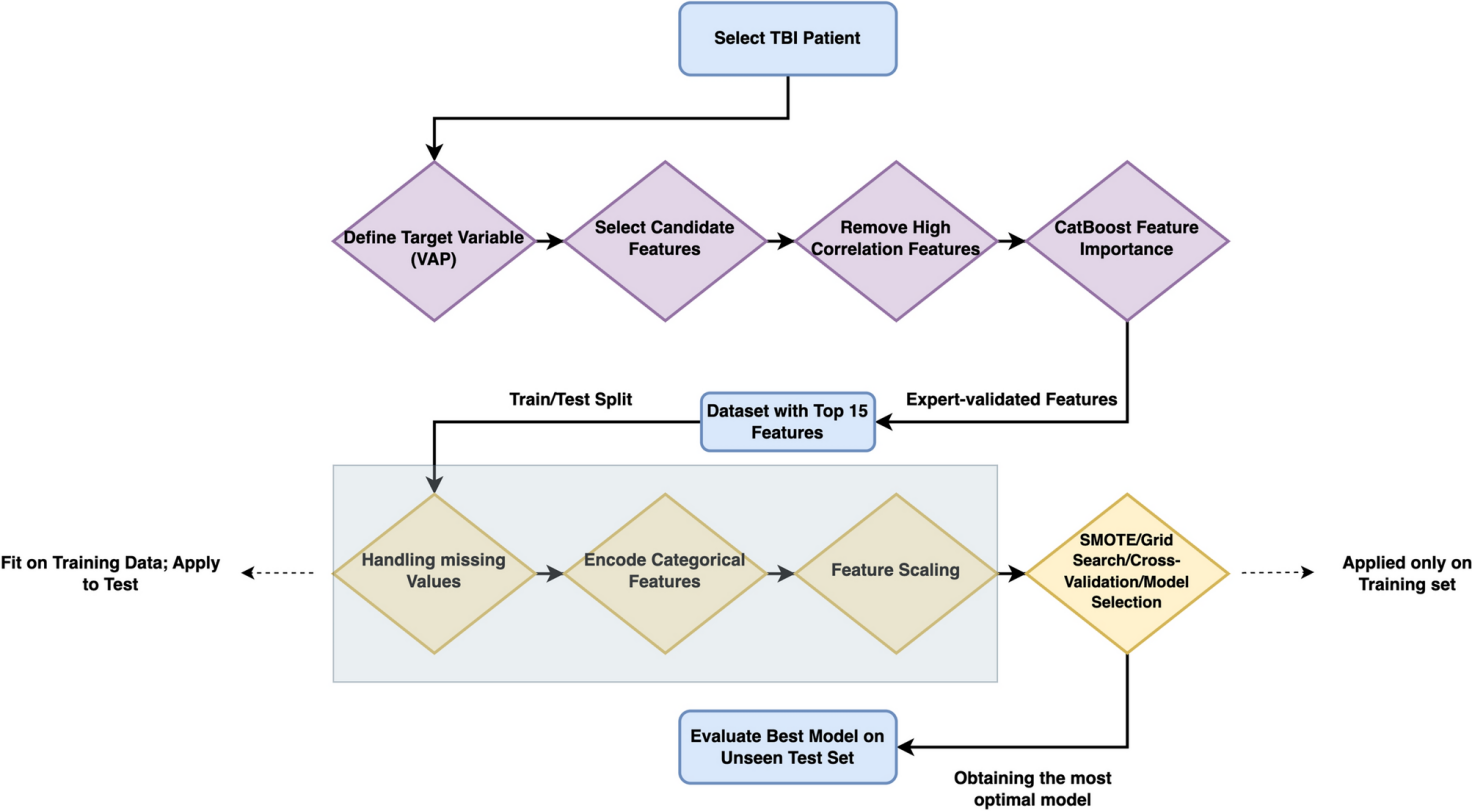
Data preprocessing workflow, illustrating the steps from patient selection to the creation of the final dataset.

To handle the class imbalance, we integrated the Synthetic Minority Over-sampling Technique (SMOTE) within a 5-fold cross-validation framework, enhancing the representation of the minority class during the training phase^25^. SMOTE was applied to the training folds only, synthesizing new samples to augment the minority (positive) class. This ensured that each training set used in the cross-validation was balanced while the original distribution of the classes was preserved in the validation folds to maintain the integrity of the validation process. By doing so, we aimed to improve the model’s generalizability and prevent data leakage, allowing us to evaluate the model’s performance against unmodified data, thus ensuring more accurate and robust outcomes. Following feature refinement (candidate selection, correlation checks, and CatBoost ranking), we split the dataset into training (70%) and testing (30%) subsets. We then fit all preprocessing transformations-such as handling missing values, encoding categorical features, and feature scaling-exclusively on the training data, applying the same transformations to the test set afterward. Subsequently, SMOTE, cross-validation, and hyperparameter tuning are carried out solely within the training partition, ensuring the test set remains completely isolated until the final model evaluation. This methodology prevents information leakage and preserves the integrity of our performance estimates. Figure 3 illustrates the workflow for data preprocessing and modeling.

### Ablation and sensitivity process

We implemented a stepwise elimination process to determine if the 15 selected features had an adverse effect on the model’s performance. This involved systematically removing variables that had an adverse effect on the model’s performance, which we measured by calculating the 95% Confidence Interval (CI) of the Area Under the Receiver Operating Characteristic Curve (AUROC). We started by calculating the baseline AUROC using all 15 features.

In each iteration, we temporarily removed one feature from the current set and recalculated the AUROC. If the AUROC for the new set of features showed improvement, we updated the current feature set by permanently excluding the feature and recorded the improved AUROC. This iterative process continued, with the model being re-evaluated after each removal, until no further improvements were observed^26^.

### Modeling

After data preprocessing, we used the top 15 important features for the modeling part. Then, we divided the dataset into training and testing sections^27^. 70% of the data was used for model training and 30% of the data for testing. Six models have been implemented in this project, including Support Vector Machine (SVM), Logistic Regression (LR), Random Forest (RF), XGBoost, Artificial Neural Network (ANN), and AdaBoost. A 5-fold cross-validation scheme is adopted to balance robust performance estimation and computational feasibility, particularly given our moderately sized dataset and ensemble-based algorithms (e.g., XGBoost). While a single train-test split may yield high variance in results, and 10-fold cross-validation could reduce variance further, the computational cost of training multiple folds on resource-intensive models can be prohibitive. Consequently, 5-fold cross-validation provides a practical middle ground.

For the SVM, we adjusted the regularization parameter, kernel type, and kernel parameters to manage model complexity and fit^28^. In LR, we tuned the regularization strength and L1 and L2 penalty types to balance bias and variance, enhancing generalizability^29,30^. The RF model was optimized by setting the number of trees, the maximum depth of the trees, and the quality criterion for splits to improve performance across different data subsets^31,32^. In the XGBoost model, adjustments included the colsample_bytree, learning rate, max_depth, min_child_weight, n_estimators, reg_alpha, reg_lambda, scale_pos_weight, and subsample to increase accuracy and prevent overfitting. The colsample_bytree parameter specifies the fraction of features to be randomly sampled for each tree, helping to prevent overfitting. The learning rate controls the step size for each boosting iteration, balancing between learning quickly and preventing overfitting. Max_depth sets the maximum depth of a tree, impacting model complexity and overfitting potential. Min_child_weight determines the minimum sum of instance weight (hessian) needed in a child, providing regularization by preventing overly specific trees. N_estimators is the number of boosting rounds. The reg_alpha and reg_lambda are L1 and L2 regularization terms, respectively, which add penalties to the model to prevent overfitting. Scale_pos_weight balances the positive and negative weights, useful for handling class imbalance. Subsample is the fraction of samples to be used for fitting the individual base learners, which helps prevent overfitting by introducing randomness. These adjustments optimize model performance and robustness against overfitting^33–35^.

The ANN consisted of an input layer, one hidden layer, and an output layer, utilizing ReLU (Rectified Linear Unit) activation functions for the input and hidden layers to facilitate non-linear learning and a sigmoid activation function in the output layer for binary classification probabilities^36,37^. Lastly, the AdaBoost model was fine-tuned by adjusting the depth of the base estimators, the learning rate, and the number of weak learners to enhance the fit and robustness of training data^38^.

From a data perspective, using metrics such as AUC and sensitivity, it is evident that the proposed model not only performs better overall but also exhibits a superior ability to predict the minority class. The model demonstrating the highest performance on the test set was selected as the best. We also assessed our models’ performance by calculating accuracy, precision, F1-score, and specificity. Details will be discussed in the results section.

## Results

### Cohort characteristics

The dataset under examination divided patients into two cohorts: the training cohort (comprising 585 patients) and the test cohort (comprising 251 patients). These cohorts were selected to validate the accuracy and reliability of clinical predictions or treatment outcomes using statistical models.

The primary aim of this analysis is to evaluate whether there are statistically significant differences between the training and test cohorts across various clinical metrics. By understanding these differences, the study aims to ensure that the training data are representative of the test data, thereby validating the model’s applicability to future, unseen patients. Table 1 illustrates a comparison of the feature values between train and testing cohorts by capturing the mean and standard deviation of features in each cohort along with the p-value associated with these two cohorts. the p-value of 0.05 is chosen as the threshold for observing significant differences between the cohorts. Table 2 compares the mean and standard deviation of 15 key features between patients who did not develop VAP (Non-VAP) and those who did (VAP). Notably, patients in the VAP group exhibit substantially longer ICU and hospital stays on average (11.651 vs. 3.286 days in ICU, and 19.513 vs. 7.245 days in total hospital stay). A higher prevalence of tracheostomy is also evident among VAP patients (0.457 vs. 0.049), suggesting more invasive interventions. Conversely, some features, such as serum sodium and anion gap, show only minor differences between groups. Overall, this comparison highlights how certain clinical and laboratory variables differ between VAP and Non-VAP populations, offering insights into the potential risk factors and severity markers associated with ventilator-associated pneumonia in TBI patients.

**Table 1:** Cohort comparison of training and test sets for all 15 features with their associated p-value.

Feature	Train mean (std)	Test mean (std)	P-value
ICU stay length	6.736 (7.152)	6.175 (6.487)	0.286
Serum potassium	3.989 (0.684)	4.008 (0.717)	0.723
Hospital stay length	12.434 (14.028)	11.182 (10.698)	0.206
Serum sodium	139.767 (4.119)	139.234 (4.381)	0.093
Blood urea nitrogen	17.800 (10.823)	17.390 (8.753)	0.596
Glucose	156.784 (50.918)	152.494 (47.112)	0.254
Anion gap	15.266 (3.589)	15.013 (3.423)	0.344
Respiratory rate	17.629 (5.075)	17.806 (3.485)	0.615
INR	1.308 (0.466)	1.402 (1.320)	0.128
Tracheostomy	0.214 (0.410)	0.199 (0.400)	0.638
Hemoglobin	12.592 (2.169)	12.501 (2.176)	0.577
Systolic BP	132.213 (14.891)	131.891 (16.211)	0.780
Heart rate	87.283 (15.525)	86.852 (15.534)	0.713
Neurosurgery	0.318 (0.466)	0.283 (0.451)	0.314
Platelet	232.297 (91.167)	238.709 (89.538)	0.349

**Table 2:** Comparison of non-VAP vs. VAP for all 15 features.

Feature	Non-VAP mean (std)	VAP mean (std)
ICU stay length	3.286 (4.685)	11.651 (6.856)
Serum potassium	3.977 (0.703)	4.022 (0.679)
Hospital stay length	7.245 (9.758)	19.513 (14.158)
Serum sodium	139.686 (4.262)	139.485 (4.116)
Blood urea nitrogen	16.839 (9.894)	18.976 (10.643)
Glucose	151.335 (47.340)	161.940 (52.857)
Anion gap	15.195 (3.585)	15.183 (3.473)
Respiratory rate	17.669 (5.147)	17.703 (3.771)
INR	1.280 (0.452)	1.422 (1.182)
Tracheostomy	0.049 (0.217)	0.457 (0.499)
Hemoglobin	12.566 (2.187)	12.563 (2.148)
Systolic BP	131.667 (15.464)	132.813 (15.014)
Heart rate	87.154 (13.782)	87.153 (17.904)
Neurosurgery	0.234 (0.424)	0.421 (0.494)
Platelet	234.328 (87.123)	234.058 (96.053)

The training and test cohorts consist of patients characterized by several clinical metrics. Notable measurements include the length of stay in the ICU and hospital, various blood chemistry markers like serum sodium, potassium, and urea nitrogen, as well as vital signs such as heart rate, and systolic blood pressure. Additional specific interventions or conditions, such as tracheostomies and neurosurgery, are also tracked.

The training cohort, larger in number, likely provides a base for developing and training predictive models, with values such as an average ICU stay of approximately 6.74 days and a mean serum sodium level of about 139.77 mmol/L. On the other hand, the smaller test cohort is utilized to evaluate the performance and generalizability of the models developed from the training cohort data. It shows slightly different averages, such as a shorter ICU stay at around 6.17 days and a serum sodium level averaging 139.24 mmol/L.

In both cohorts, metrics such as glucose level and heart rate are monitored to gauge the patient’s overall health and immediate medical needs. These measurements, along with others, contribute to a comprehensive profile of each patient, which is crucial for accurate modeling and subsequent decision-making in a clinical setting.

### Ablation and sensitivity study

**Fig. 4:**
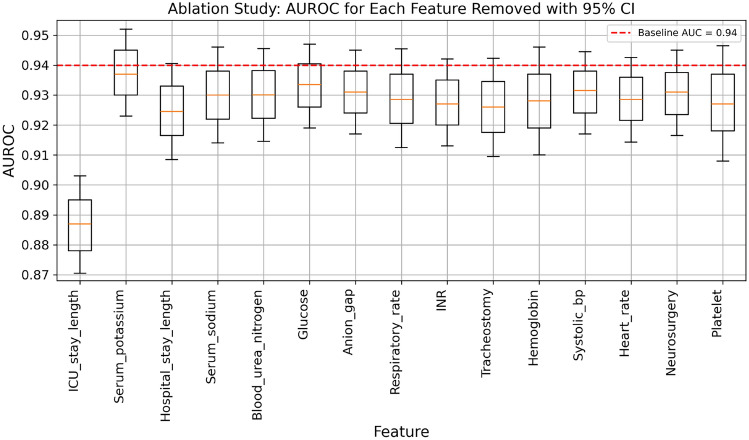
Impact of feature removal on XGBoost model performance.

The results of our ablation study, comprehensively summarized in Fig. 4, provide a clear and detailed analysis of the model’s performance with varying feature sets. After removing each feature, we run several times to get different values of AUROC and calculating its interval. The line inside each box is the median, which splits the AUROC values so that half of them lie above and half lie below. The top edge of the box is the 75th percentile, meaning 75% of the AUROC values are at or below this level, while the bottom edge is the 25th percentile, meaning 25% of the values are at or below that level. The box itself thus encloses the middle 50% of the data. The whiskers extend to 1.5 times the interquartile range (the difference between the 75th and 25th percentiles), capturing most of the remaining values. This figure suggests that the inclusion of all features is crucial, as any attempt to remove even a single feature results in a noticeable drop in the AUROC. Specifically, the figure demonstrates that the removal of any feature does not lead to an increase in AUROC; rather, it leads to a reduction, indicating that each feature contributes uniquely to the model’s predictive accuracy. The baseline model, which incorporates all 15 features, achieves a commendable AUROC of 0.94 (95% CI 0.935–0.954). The study further reveals that no feature removal leads to an AUROC value higher than this benchmark. This finding underscores the integral role of the current feature set in maintaining the model’s optimal performance, thereby negating the necessity for any further deletions or adjustments of features. The robustness and reliability of the baseline model are thus validated, highlighting the synergistic effect of the included features in enhancing the model’s predictive capability.

Beyond the drop in AUROC, removing each individual feature also diminished accuracy, F1 score, and other performance measures, underscoring the unique contribution of every predictor. Notably, omitting ICU stay length caused the steepest decline in AUROC, indicating its critical importance for detecting VAP risk. Collectively, these findings confirm that the final 15-feature set provides a complementary balance of clinical indicators essential for maintaining strong predictive performance.

### Evaluation results

To optimize the performance of our XGBoost model, a grid search was employed to identify the best hyperparameters. The grid search process systematically explored a range of values for each parameter to find the combination that yielded the highest model performance on the training data. The optimal parameters identified through this process were: a colsample_bytree value of 0.7, a learning_rate of 0.01, a max_depth of 5, a min_child_weight of 5, n_estimators set to 300, reg_alpha set to 0.1, reg_lambda set to 2, a scale_pos_weight of 2, and a subsample value of 0.7.

The results of our study emphasize the predictive performance of various machine learning techniques for VAP in patients with TBI, focusing primarily on the test set. The evaluation metrics are AUC, Accuracy, F1 Score, Specificity, Sensitivity, Positive Predictive Value (PPV), and Negative Predictive Value (NPV). The XGBoost algorithm emerged as the most effective model.

**Fig. 5:**
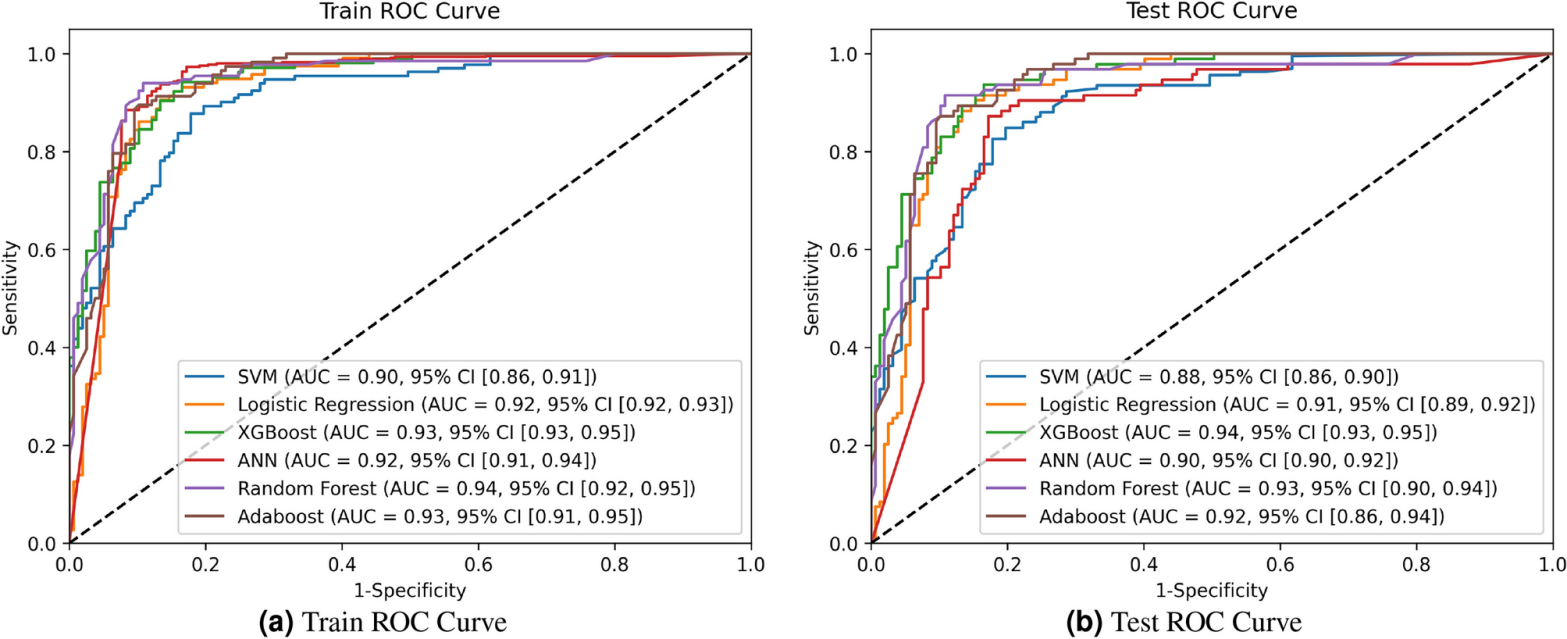
ROC curves of the six models for both the training and test sets. The models include SVM, LR, XGBoost, RF, ANN, and AdaBoost.

As shown in Table 3, the performance of the models on the training set was initially assessed, with RF achieving the highest AUC of 0.94. This was further illustrated by the Receiver Operating Characteristic (ROC) curves in Fig. 5, which highlight RF’s exceptional performance on the training data, followed closely by XGBoost with an AUC of 0.935.

However, the true test of a model’s generalizability is its performance on unseen data. As shown in Table 3, XGBoost achieved the highest AUC of 0.940 with a 95% Confidence Interval (CI) [0.935-0.954] on the test set, demonstrating superior overall performance compared to other models. This was further validated by the ROC curves depicted in Fig. 5, illustrating XGBoost’s robust discriminative ability. Additionally, XGBoost maintained a high accuracy of 0.875, sensitivity of 0.896, and specificity of 0.857, indicating its balanced performance across different evaluation metrics.

**Table 3:** Predictive performance of machine learning models for VAP among TBI patients in the training and test sets.

Models	AUC (95% CI)	Accuracy (95% CI)	F1 Score (95% CI)	Sensitivity (95% CI)	Specificity (95% CI)	PPV (95% CI)	NPV (95% CI)
Training set							
SVM	0.904 (0.862–0.914)	0.857 (0.823–0.891)	0.787 (0.741–0.833)	0.773 (0.704–0.843)	0.916 (0.872–0.961)	0.812 (0.751–0.874)	0.888 (0.854–0.923)
LR	0.921 (0.924–0.931)	0.897 (0.883–0.910)	0.834 (0.812–0.856)	0.795 (0.763–0.827)	0.958 (0.954–0.961)	0.878 (0.868–0.888)	0.906 (0.890–0.922)
XGBoost	0.935 (0.935–0.955)	0.925 (0.911–0.938)	0.892 (0.876–0.908)	0.946 (0.927–0.965)	0.907 (0.878–0.935)	0.842 (0.811–0.873)	0.983 (0.972–0.994)
ANN	0.924 (0.914–0.943)	0.895 (0.887–0.903)	0.844 (0.831–0.856)	0.843 (0.827–0.859)	0.926 (0.916–0.935)	0.843 (0.828–0.858)	0.926 (0.917–0.935)
RF	0.940 (0.920–0.951)	0.895 (0.848–0.942)	0.856 (0.801–0.912)	0.887 (0.798–0.976)	0.883 (0.805–0.961)	0.811 (0.730–0.893)	0.953 (0.906–1.0)
AdaBoost	0.932 (0.914–0.951)	0.907 (0.883–0.930)	0.866 (0.844–0.898)	0.885 (0.835–0.965)	0.915 (0.872–0.958)	0.843 (0.798–0.888)	0.951 (0.911–0.992)
Test set							
SVM	0.882 (0.862–0.903)	0.807 (0.773–0.841)	0.737 (0.691–0.783)	0.723 (0.654–0.793)	0.866 (0.822–0.911)	0.762 (0.701–0.824)	0.838 (0.804–0.873)
LR	0.914 (0.893–0.924)	0.847 (0.833–0.860)	0.784 (0.762–0.806)	0.745 (0.713–0.777)	0.908 (0.904–0.911)	0.828 (0.818–0.838)	0.856 (0.840–0.872)
XGBoost	0.940 (0.935–0.954)	0.875 (0.861–0.888)	0.842 (0.826–0.858)	0.896 (0.877–0.915)	0.857 (0.828–0.885)	0.792 (0.761–0.823)	0.933 (0.922–0.944)
ANN	0.896 (0.895–0.921)	0.845 (0.837–0.853)	0.794 (0.781–0.806)	0.793 (0.777–0.809)	0.876 (0.866–0.885)	0.793 (0.778–0.808)	0.876 (0.867–0.885)
RF	0.925 (0.896–0.935)	0.845 (0.798–0.892)	0.806 (0.751–0.862)	0.837 (0.748–0.926)	0.833 (0.755–0.911)	0.761 (0.680–0.843)	0.903 (0.856–0.949)
AdaBoost	0.918 (0.861–0.936)	0.857 (0.833–0.880)	0.816 (0.784–0.848)	0.835 (0.755–0.915)	0.865 (0.822–0.908)	0.793 (0.748–0.838)	0.901 (0.861–0.942)

Other model evaluations include RF, LR, ANN, SVM, and AdaBoost. The RF, despite having the highest training set AUC, showed a lower AUC value of 0.925 in the test set, highlighting the importance of evaluating models on unseen data to ensure their generalizability. ANN, LR, and AdaBoost also showed highly competitive results with AUCs 0.896, 0.914, and 0.918, respectively, but their performance did not surpass that of XGBoost. SVM demonstrated moderate performance with an AUC of 0.882.

**Fig. 6:**
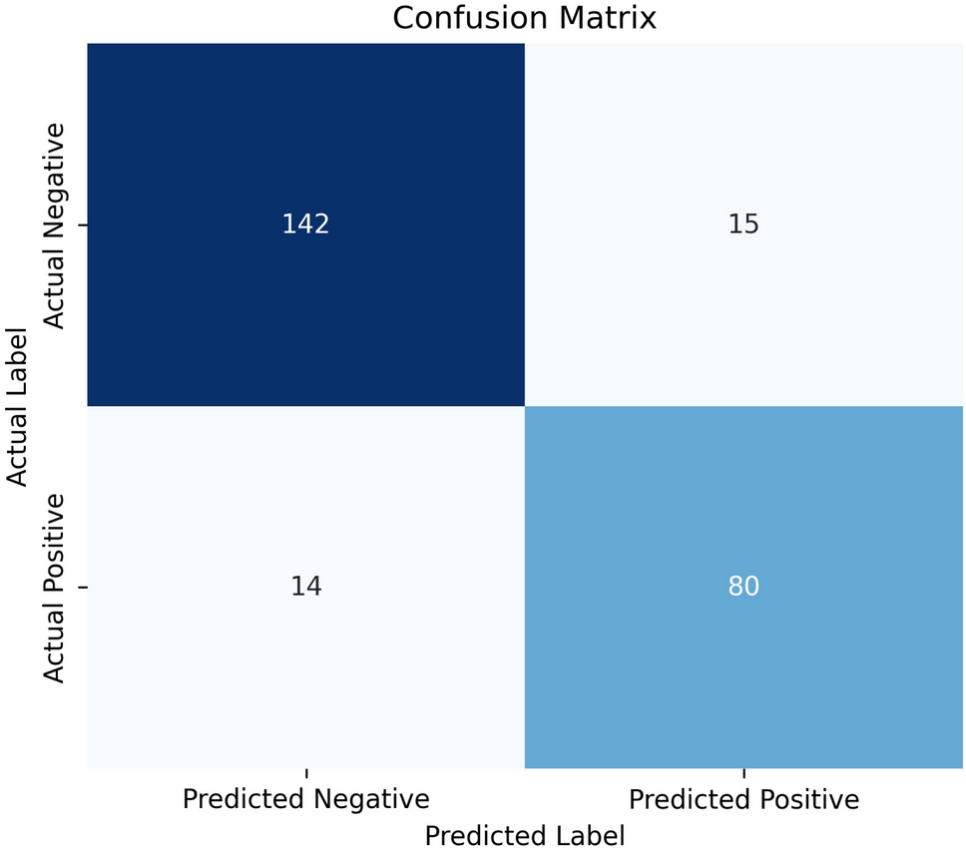
Confusion matrix analysis.

Figure 6 presents the confusion matrix of our best-performing model on the test set. From these counts, we can see that the model correctly identifies a substantial portion of both positive and negative cases, as indicated by the relatively low false-positive (15) and false-negative (14) counts. This balance is crucial in clinical applications where misclassifying either class (especially false negatives) can have serious consequences.

The clear advantage of XGBoost in the test cohort highlights its efficacy in handling the complexities and nuances associated with predicting VAP in TBI patients. This model’s ability to maintain high sensitivity and specificity makes it particularly suitable for clinical applications where accurate identification of at-risk patients is crucial. The findings suggest that implementing XGBoost in predictive analytics for healthcare could significantly enhance decision-making processes and patient outcomes.

### SHAP feature importance

**Fig. 7:**
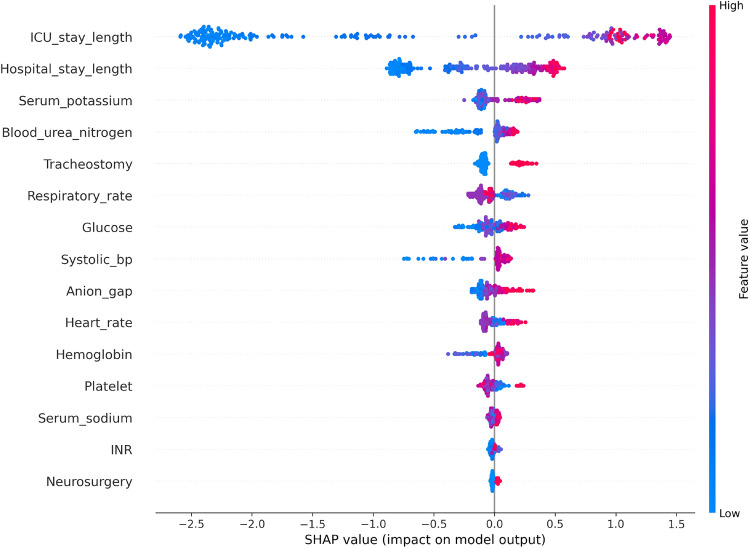
SHAP values for the test set, derived from the XGBoost model.

The SHAP summary plot is demonstrated in Fig. 7. It provides a detailed understanding of the features impacting the prediction model for VAP in patients suffering from TBI. Notably, ICU length of stay and hospital length of stay are the most influential features as also shown in^39^.

Longer ICU stays significantly decrease the model’s predicted output, suggesting a higher risk or poorer outcome, while longer hospital stays also decrease the output but to a lesser extent. High serum potassium levels have a slight positive impact on the model’s predictions, whereas high blood urea nitrogen levels show a varied but generally positive effect on the output. The analysis also reveals that features like respiratory rate and glucose levels, when elevated, positively influence the model’s predictions, indicating better outcomes or lower risk. The presence of a tracheostomy shows mixed but generally positive impacts. The non-linear relationships captured by the model highlight the complexity of interactions between features and outcomes. This insight is crucial for improving early detection and interventions for VAP in TBI patients, enhancing model interpretability and clinical applicability. Compared to^5^, our SHAP analysis provides a more detailed and nuanced understanding of feature importance. The SHAP summary plot in our study reveals a greater complexity and depth, highlighting the influence of features such as ICU stay length, hospital stay length, serum potassium, and blood urea nitrogen on the model’s predictions. This level of detail offers a clearer insight into the factors contributing to VAP development, enhancing the interpretability and reliability of our predictive models.

## Discussion

### Summary of existing model compilation

In our study, XGBoost demonstrated better performance compared to other models. This advantage can be explained by the sophisticated mechanisms this model employs. Firstly, XGBoost employs ensemble learning techniques that combine multiple weak learners to form a strong learner, inherently reducing variance and bias for more robust and accurate models. Additionally, these models are particularly effective in handling imbalanced data, a common challenge in medical diagnostics, by optimizing the classification boundary better than simpler models that often struggle with minority class predictions. XGBoost also excels in managing different data types and missing values, and it incorporates built-in regularization to minimize overfitting. XGBoost also incorporates regularization techniques such as L1 (Lasso) and L2 (Ridge) regularization.

However, it is important to note that LR showed better performance in terms of specificity and PPV. The specificity of LR indicates its higher ability to correctly identify negative cases, reducing the number of false positives, which is crucial in medical diagnostics to avoid unnecessary treatments. Additionally, the higher PPV of LR reflects its greater precision in predicting positive cases, which ensures that the identified cases are more likely to be true positives. Nonetheless, since XGBoost performed better in other metrics, we selected it for our proposed model.

### Comparison with literature results

A related study by Wang et al. also used the MIMIC-III database to identify TBI patients, but employed a substantially larger feature set than our 15 features^5^. This larger number of features likely contributed to severe overfitting in their models on the training set. Their approach included seven different models: SVM, LR, RF, Light Gradient Boosting Machine (Light GBM), AdaBoost, Multilayer Perceptron (MLP), and XGBoost. In contrast, we included ANN in our study, which they did not utilize. We decided against using Light GBM and MLP after observing suboptimal performance with these models in the original research. The inclusion of ANN was to assess the efficacy of deep learning in our context, although it did not yield satisfactory results.

The previous study provided a table of metrics for each model rather than presenting a single best model. Here, we confirm that our proposed model surpasses every model from the previous study in each metric by comparing our results with the best metrics reported for those models.

Our proposed XGBoost model achieved an AUC of 0.940 (95% CI 0.935–0.954), outperforming the previous study’s AdaBoost model’s best AUC of 0.706 (95% CI 0.624–0.788) by 0.234, indicating superior discrimination capability. The model’s accuracy was 0.875 (95% CI 0.861–0.888), a 0.235 improvement over the previous study’s XGBoost accuracy of 0.640 (95% CI 0.616–0.663), reflecting enhanced predictive performance. Sensitivity reached 0.896 (95% CI 0.877–0.915), up by 0.204 from the previous study’s XGBoost sensitivity of 0.692 (95% CI 0.628–0.756). Specificity was 0.857 (95% CI 0.828–0.885), slightly higher than the previous Light GBM model’s 0.849 (95% CI 0.815–0.883). The Positive Predictive Value (PPV) improved to 0.792 (95% CI 0.761–0.823), up by 0.084 from the previous RF model’s 0.708 (95% CI 0.615–0.801). The Negative Predictive Value (NPV) was 0.933 (95% CI 0.922–0.944), surpassing the previous AdaBoost model’s 0.706 (95% CI 0.686–0.726) by 0.227. Our F1 Score reached 0.842 (95% CI 0.826–0.858), an improvement of 0.182 over the previous RF model’s 0.660 (95% CI 0.571–0.748). The ROC curves in Fig. 5 further validate our approach.

Our approach addresses key gaps in the prior literature, specifically the lack of feature selection and class imbalance handling. We reduced multicollinearity by removing highly correlated features and selected the top 15 relevant features based on CatBoost importance scores, verified by a clinical expert to ensure model robustness and predictive power. To address class imbalance, a critical issue in medical datasets, we applied SMOTE to synthesize new examples in the minority class, resulting in a balanced dataset. This step improved the generalizability and fairness of our predictive models.

Our results demonstrate superior performance compared to existing literature, with significant improvements in AUC, accuracy, and other metrics. A higher AUC indicates our model’s improved consistency in distinguishing patients at risk for VAP, enhancing predictive capability for timely clinical interventions. Additionally, the increased sensitivity reflects a better ability to identify positive cases, which is crucial for medical diagnostics. Feature importance analysis (Fig. 7) reveals that ICU stay length, hospital stay length, serum potassium, blood urea nitrogen, and tracheostomy were the most influential features. In contrast, prior studies identified tracheostomy, RBC transfusion, and PEG as top factors in their AdaBoost models. These differences highlight our model’s ability to more accurately capture the critical factors impacting VAP development.

### Study limitations

Firstly, our analysis relies solely on the MIMIC-III database. Although comprehensive, this dataset is over a decade old and may not capture current clinical practices or the most recent patient demographics. Future research should validate our findings using more recent datasets like MIMIC-IV or other contemporary repositories. Additionally, integrating multimodal data-including textual clinical notes, imaging, or even genomic data-could provide a richer representation of patient profiles and further improve the predictive accuracy for VAP.

Another limitation concerns our approach to handling class imbalance. While we employed SMOTE within a 5-fold cross-validation framework, SMOTE may introduce synthetic samples that do not fully capture the complex variability of real-world clinical data. Future studies could explore alternative methods such as ensemble techniques, cost-sensitive learning, or generative adversarial networks (GANs) to better address the imbalance and improve model robustness.

Our current modeling framework also overlooks temporal dependencies in ICU patient data. VAP and other clinical conditions evolve over time, and static models might fail to capture these dynamic changes. Incorporating time-series analysis or using architectures such as recurrent neural networks (RNNs) could help model patient trajectories more effectively, potentially leading to earlier and more accurate predictions.

Additionally, although our retrospective analysis demonstrates high predictive performance, the practical implementation of our model in a clinical setting remains untested. Future work should focus on integrating the model into electronic health record (EHR) systems and evaluating its performance in prospective clinical trials. Collaborations with clinicians and health informatics experts will be crucial to develop user-friendly interfaces and ensure that the model’s outputs are actionable in real-world scenarios.

Lastly, while our study includes rigorous hyperparameter tuning and ablation studies, the risk of overfitting cannot be completely ruled out, especially given the retrospective nature of the analysis. External validation across multiple centers and prospective studies are needed to further assess the generalizability of our model.

In summary, updating and diversifying the data sources, adopting more sophisticated methods for class imbalance, incorporating temporal dynamics, and performing prospective clinical validations are essential next steps to enhance the accuracy, generalizability, and practical utility of predictive models for ventilator-associated pneumonia in traumatic brain injury patients.

## Conclusion

This study systematically addressed the challenge of predicting ventilator-associated pneumonia in traumatic brain injury patients by applying a meticulous end-to-end methodology. Our workflow began with patient selection from the MIMIC-III database, followed by data cleaning and missing-value imputation. We then carefully reduced an initial set of 52 features down to 15 through correlation checks and CatBoost importance scores, validated by clinical expertise. This refined feature set helped alleviate multicollinearity and focused our models on the most clinically relevant predictors, such as ICU stay length, hospital stay length, serum potassium, and blood urea nitrogen.

To counteract the inherent class imbalance, we employed SMOTE within a rigorous 5-fold cross-validation protocol, ensuring a more equitable representation of minority-class (VAP) cases. We subsequently trained and compared six machine learning models-Support Vector Machine, Logistic Regression, Random Forest, XGBoost, Artificial Neural Network, and AdaBoost-tuning their hyperparameters extensively. Notably, our ablation study confirmed that excluding any selected feature compromised the model’s performance, emphasizing the necessity of the chosen feature set.

Among all models, XGBoost emerged as the top performer, achieving an AUC of 0.94 and an accuracy of 0.875, significantly outperforming earlier studies that reported an AUC of 0.706 and accuracy of 0.640. This improvement was further corroborated by higher sensitivity (0.896), specificity (0.857), Positive Predictive Value (0.792), Negative Predictive Value (0.933), and F1-score (0.842) compared to previous benchmarks. Additionally, SHAP analysis provided valuable insights into feature importance, illustrating how variables such as ICU stay length and serum potassium influenced predictions. This enhanced interpretability is critical for clinical adoption, as it offers transparency and confidence in the model’s decision-making process.

These findings hold substantial clinical relevance. Early and accurate VAP detection in TBI patients can guide more proactive interventions, reduce complication-related mortality, and streamline resource allocation. Future work could validate the model using more contemporary datasets (e.g., MIMIC-IV) or real-time clinical data, explore additional predictive features like imaging or free-text notes, and integrate the model into bedside decision-support systems. Such efforts would further bolster our framework’s applicability, enabling evidence-based, high-precision care in critical settings.

## Data Availability

The raw dataset is available in the MIMIC-III repository: https://physionet.org/content/mimiciii/1.4/.
